# Seed-Specific Expression of Spider Silk Protein Multimers Causes Long-Term Stability

**DOI:** 10.3389/fpls.2016.00006

**Published:** 2016-01-28

**Authors:** Nicola Weichert, Valeska Hauptmann, Christine Helmold, Udo Conrad

**Affiliations:** Department of Molecular Genetics, Leibniz Institute of Plant Genetics and Crop Plant ResearchGatersleben, Germany

**Keywords:** seed expression, spider silk proteins, intein, protein *trans*-splicing, tobacco

## Abstract

Seeds enable plants to germinate and to grow in situations of limited availability of nutrients. The stable storage of different seed proteins is a remarkable presumption for successful germination and growth. These strategies have been adapted and used in several molecular farming projects. In this study, we explore the benefits of seed-based expression to produce the high molecular weight spider silk protein FLAG using intein-based *trans*-splicing. Multimers larger than 460 kDa in size are routinely produced, which is above the native size of the FLAG protein. The storage of seeds for 8 weeks and 1 year at an ambient temperature of 15°C does not influence the accumulation level. Even the extended storage time does not influence the typical pattern of multimerized bands. These results show that seeds are the method of choice for stable accumulation of products of complex transgenes and have the capability for long-term storage at moderate conditions, an important feature for the development of suitable downstream processes.

## Introduction

Seeds have evolved because of their unique properties, providing important features to plants to survive and to propagate even under harsh environmental conditions. Seeds enable plants to germinate and to grow in situations of limited availability of nutrients. The presence of storage products in seeds is necessary for their functionality. Among a plethora of compounds of different classes, storage proteins play an important role. Throughout long periods of dormancy, storage proteins remain intact (Golovina et al., 1997). Seeds and their compartments contribute to the competiveness of different plant species. The stable storage of different seed proteins is a remarkable presumption (Boothe et al., 2010). These strategies have been adapted and used in several molecular farming projects [for review, see Stöger et al. (2005)]. Recombinant antibodies, i.e., single chain Fv antibodies, have been produced in tobacco seeds under the control of a seed-specific faba bean legumin promoter (Fiedler and Conrad, 1995). Storage protein promoters, as well as other seed-specific promoters, such as the USP promoter in dicots, are well suited for seed-based production combined with ER retention (Fiedler et al., 1997). These seeds can be stored for at least 1 year without loss in the amount and activity of the transgenic protein (Fiedler and Conrad, 1995). Stable storage at ambient conditions is an important feature, because it allows the development of harvesting/downstream processing strategies that do not need a long-term cooling chain or a direct production/extraction/purification process.

Spider-silk proteins have been a target of molecular farming since 2001 (Scheller et al., 2001). The enormous interest in this biopolymer is caused by its extraordinary properties, such as high levels of toughness, tensile strength, and elasticity (Vollrath and Knight, 2001; Craig, 2004). The capture spiral silk, also called flagelliform silk, can be stretched extremely far before rupture. This high level of elasticity is required to ensure prey capture (Vollrath and Edmonds, 1989; Köhler and Vollrath, 1995). The perfect dissipation of kinetic impact energy of flying prey is a requirement for capture fibers to withstand the relative high velocity of flying prey (Römer and Scheibel, 2008). The flagelliform silk consists of only one protein, called FLAG. The elastic properties are thought to be based on the presence of a GPGGX consensus motif in a high number of repeats. Helical GGX repeats are also typical consensus repeats of this protein (Hayashi et al., 1999; Hayashi and Lewis, 2000). These two consensus elements together are responsible for the elasticity and flexibility of the flagelliform silk (Ohgo et al., 2006). A common feature of all known silk proteins from the *arthropoda* is a high molecular weight of more than 250 kDa. This evolutionary convergence between unrelated species is striking. Therefore, the enormous size of all these proteins is anticipated as a necessary prerequisite for the extraordinary mechanical properties (Lewis, 2006). In spider silk proteins, motifs conducive for inter- and intra-chain interactions are common, and chain end defects are rare events (Ayoub et al., 2007). The recombinant production of spider silk proteins in pro- and eukaryotic expression systems limits the maximal size of these proteins. Here, the genetic instability of these highly repetitive proteins and the limited availability of frequently used amino acids, as well as the corresponding t-RNAs, are possible reasons. Synthetic spider silk proteins of native size can only be produced by a metabolically modified *Escherichia coli* strain (Xia et al., 2010). More slowly growing organisms, such as plants, are used to overcome these protein size limitations associated with t-RNA and amino acid availability. The maximal size achieved was approximately 100 kDa (Scheller et al., 2001, 2004). Post-translational multimerization methods have been chosen to further increase the size of recombinant spider silk protein derivatives. Purified spider silk-ELP fusion proteins from tobacco leaves were enzymatically multimerized by transglutamination. Layers formed by highly cross-linked spider silk-ELP fusion proteins were associated with a high elastic indentation modulus and, therefore, higher toughness and stiffness of layers formed by multimerized plant-based spider silk protein derivatives were expected (Weichert et al., 2014). We developed a general system for the production of highly repetitive proteins in plants. Protein *trans-*splicing by inteins was used to assemble protein subunits *in planta* (Yang et al., 2003; Kempe et al., 2009); for review see Evans et al. (2005). Inteins are autocatalytically excised from precursor proteins and fuse the flanking exteins together (Perler, 1998). A few of inteins from bacteria have been described (Perler et al., 1994). An intein from cyanobacteria (Pietrokovski, 1996) has been demonstrated to function in plants (Evans et al., 2000; Yang et al., 2003; Kempe et al., 2009). A comprehensive description of *in vivo* applications of intein-mediated protein splicing is given by Topilina and Mills (2014). Hauptmann et al. (2013) demonstrated that multimers of at least the native size of the spider silk protein FLAG could be produced by intein-based *trans*-splicing and purified from tobacco leaves. Purified and desalted FLAG multimers formed microfibers after drying, thus demonstrating their potential as future biomaterials.

Several applications of spider-silk-derived biopolymers in the field of engineering and technology are discussed in the literature (Kluge et al., 2008; Hardy and Scheibel, 2009). A possible medical application is the use of spider silk particles for the controlled delivery of protein drugs (Hofer et al., 2012). The Ancient Greeks used cobwebs for wound healing when covering bleeding lesions (Gerritsen, 2002). Spider silks can enhance axonal regeneration (Radtke et al., 2011), serve as a scaffold for human cell growth (Widhe et al., 2010), and support the proliferation of fibroblasts and keratinocytes (Wendt et al., 2011). Cytocompatibility is an important prerequisite for any medical use of biomaterials. A plant-produced synthetic spidroin fused with a hundred repeats of elastin-like-peptides (ELP) has been shown to be non-toxic and to enhance the proliferation of human chondrocytes and prevent dedifferentiation (Scheller et al., 2004). Cytocompatibility assays with plant-produced spidroin-ELP biopolymers gave no indication of spidroin-derived cytotoxicity, and no hemolytic effects have been detected (Hauptmann et al., 2015).

In the present paper, we questioned whether the benefits of seed-based production could be extended to the production of high molecular weight spider silk proteins. High molecular weight spider silk proteins could have superior mechanical properties combined with non-cytotoxic and non-hemolytic behavior. We also considered whether intein-based *trans*-splicing also functions in seeds, and we demonstrated that spider silk proteins of native size could be produced in seeds.

## Materials and Methods

### Construct Design

The 1149 bp *unknown seed protein* (*usp)* promoter (Zakharov et al., 2004) was PCR-amplified using 5′-CGAGTCGACATTTTTACATGATATAATG-3′ and 5′-CGTCCATGGACTGGCTATGAAGAAATTATAATC-3′ primers. The resulting PCR product was introduced into the *Hin*cII and *Nco*I restriction sites of a pRTRA 15-based plasmid described by Hauptmann et al. (2013). This plasmid contained the complete *IntC::Flag::IntN* gene construct, including the *LeB4* legumin signal peptide, ER retention signal KDEL, the c-myc-tag and the *CaMV35S* terminator. The synthetic *InteinC::Flag::InteinN (IntC::Flag::IntN)* gene construct was based on *Flag* gene motifs from publicly available *Nephila clavipes* cDNAs (GenBank accession nos. AF027972 and AF027973) and the Intein-encoding sequence from *Synechocystis* sp. gene *DnaB* (UniProtKB/Swiss-Prot accession no. Q55418; Hauptmann et al., 2013). The complete *Flag* expression cassette (USP-FIC) was inserted into the binary vector pCB301-Kan (Xiang et al., 1999; Scheller et al., 2001) via the *Hin*dIII site, resulting in the expression plasmid USP-FIC/pCB301-Kan.

### Production of FLAG Overexpressing Plants

The binary plasmid USP-FIC/pCB301-Kan was transformed into the *Agrobacterium tumefaciens* strain C58C1 (pGV2260; Deblaere et al., 1985) by electroporation. For stable transgene expression in two different tobacco varieties, *Nicotiana tabacum* cv. Samsun NN (SNN) and *N. tabacum* cv. Petit Havana, plants were transformed by agroinfection based on the leaf-disk method (Horsch et al., 1985) and elaborated by Floss and Conrad (2010). Tobacco leaf disks were submerged for 1 h in overnight-grown, liquid, *Agrobacterium* culture and plated on Murashige-Skoog (MS) agar for 2 days at 24°C in the dark. Infected explants were transferred to NBKC agar (MS medium containing 0.2 mg/L α-naphthalene acetic acid, 1 mg/L 6-benzylaminopurine, 50 mg/L kanamycin, and 500 mg/L cefotaxime). Every 10–14 days, the plantlets were transferred to fresh NBKC agar until differentiation. The developing transgenic plants were cultured on MS agar containing 50 mg/L kanamycin and were selected by immunoblotting using an anti-c-myc antibody (Evan et al., 1985). Recombinant protein expressing plants were grown in greenhouses to maturity for further propagation. Seeds were analyzed by anti-c-myc immunoblotting for overexpression of the target proteins.

### Seed Material

Mature tobacco seeds, as well as developing seeds at defined developmental stages [18 days after flowering (DAF), 21 DAF], were harvested. Immature seed material was immediately frozen in liquid nitrogen and stored at -80°C. Mature seed material was stored at 15°C with 49% humidity.

### SDS-PAGE and Immunoblotting Analysis

For analysis of transgenic plants, seed material was ground in seed extraction buffer (50 mM Tris pH 8.0, 200 mM NaCl, 5 mM EDTA, 0.1% Tween). SDS sample buffer (Gahrtz and Conrad, 2009) was added in a 1:1 ratio. The homogenate was incubated at 95°C for 10 min and was cleared by centrifugation (30 min, 4°C, 12,000 rpm). The total protein content of the supernatant was determined using the Bradford assay (Bio-Rad, Germany). Seed extracts were separated by reducing SDS-PAGE (3 or 4–10% polyacrylamide gradient), were electrotransferred to a nitrocellulose membrane and immunodetection was performed as described by Conrad et al. (1998) using anti c-myc antibodies (Evan et al., 1985). Tobacco seed proteins were separated by SDS-PAGE and stained by Coomassie Brilliant Blue R-250 (SERVA GmbH, Germany). The accumulation analysis in a semiquantitative manner was done by help of different concentrations of an anti-TNF-V_H_H-100xELP standard (Conrad et al., 2011). One c-myc tag is connected with 72 kDa protein, whereas in all FLAG multimers one c-myc tag is always connected with 37.6 kDa protein (Hauptmann et al., 2013). A FLAG multimer band corresponding to a standard band, therefore, always corresponds to about half of the protein amount of the standard band. We roughly estimated the FLAG content by counting the corresponding bands according the different standard amounts and summarized the results for every lane. Extracts from 200 seeds were separated in each lane. We separated extracts from given numbers of seeds per lane, estimated the fresh weight per seed (70 μg per seed for cv. Samsun NN and 65 μg per seed for cv. Petit Havana) and calculated the transgenic protein per fresh weight.

## Results

### FLAG Multimers are Stably Accumulated in Tobacco Seeds

**FIGURE 1:**
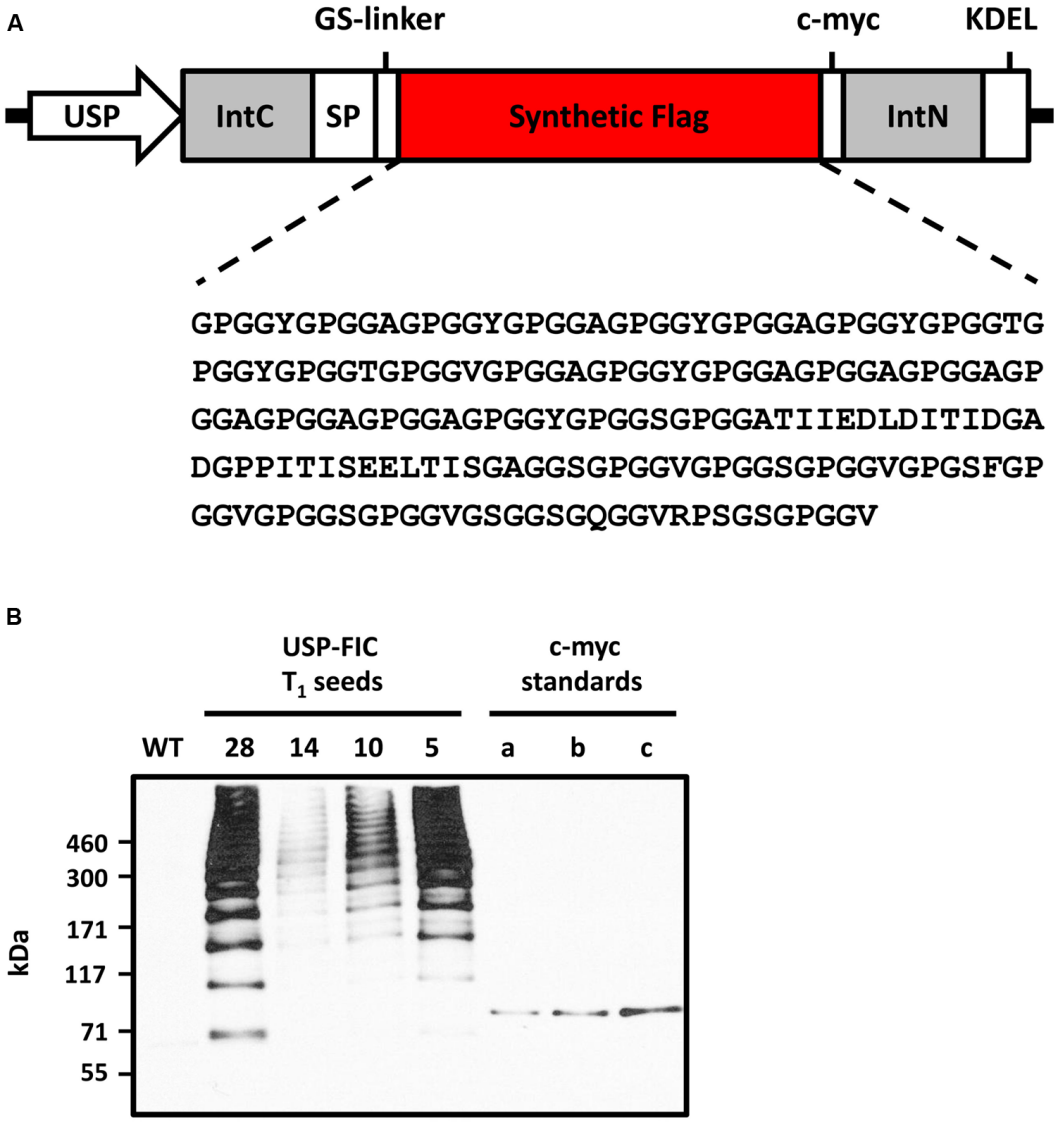
**Expression of synthetic FLAG multimers in tobacco seeds. (A)** Schematic representation of the plant expression cassette for seed-specific expression of intein-based assembled high molecular weight synthetic FLAG. Abbreviations: USP, unknown seed protein promoter; SP, legumin B4 signal peptide; KDEL, ER retention sequence; IntC/IntN, C- and N-terminal intein sequences of the *Synechocystis* sp. DnaB; GS-linker, flexible (GGGGS)_3_ spacer; c-myc, immunodetection tag. **(B)** Extracts of T_1_ seeds of four different transformed tobacco plants expressing the FLAG-Intein-c-myc protein (USP-FIC) and of corresponding wild type cultivar *Nicotiana tabacum* cv. Samsun NN were separated by gradient SDS-PAGE (4–10% PAA) with 30 μg total soluble seed protein loaded per lane reflecting 4.1 seeds (USP-FIC 28), 4.0 seeds (USP-FIC 14), 4.7 seeds (USP-FIC 10), and 3.9 seeds (USP-FIC 5). FLAG multimers were immunodetected by Western blotting based on the c-myc tag. c-myc standards (a) 0.5 ng, (b) 1 ng, (c) 2 ng of anti-TNF-V_H_H-100xELP (Conrad et al., 2011); WT, wild type.

**FIGURE 2:**
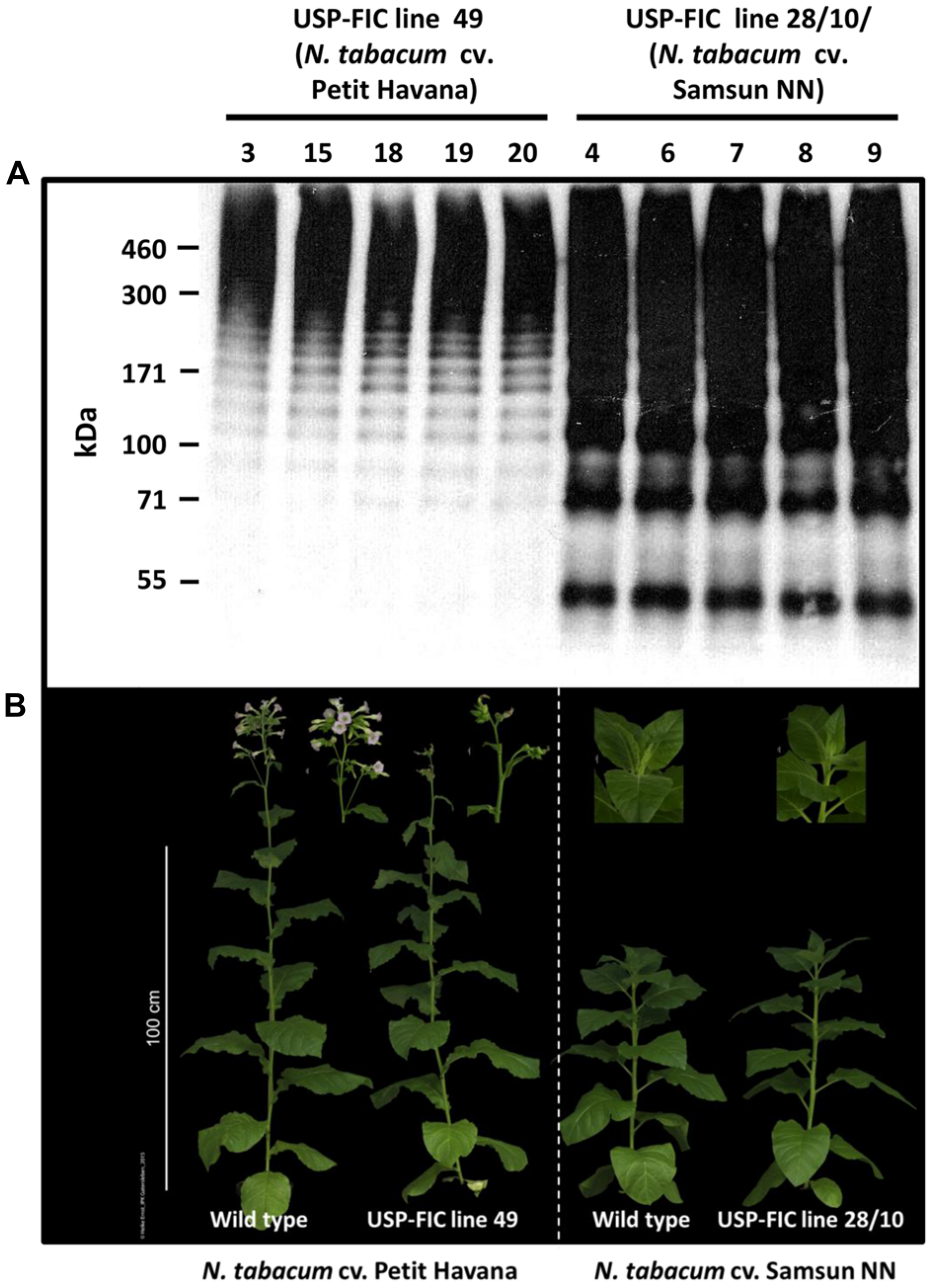
**Accumulation of FLAG multimers in seeds of two different tobacco varieties. (A)** Analysis of ripe T_3_ seeds of *N. tabacum* cv. SNN USP-FIC line 28/10 and T_2_ seeds of *N. tabacum* cv. Petit Havana USP-FIC line 49 by gradient SDS-PAGE (4–10% PAA) with 20 μg total soluble seed protein per lane, Western blotting and immunodetection based on the c-myc tag; kDa, kilodalton. **(B)** Transgenic USP-FIC line 49 with a genomic background of *N. tabacum* cv. Petit Havana as well as the corresponding wild type plants showed a faster vegetative growth and flowered 8 days earlier than Samsun NN-genome-based FLAG overexpressing plants of USP-FIC line 28/10 and its corresponding wild type plants.

**FIGURE 3:**
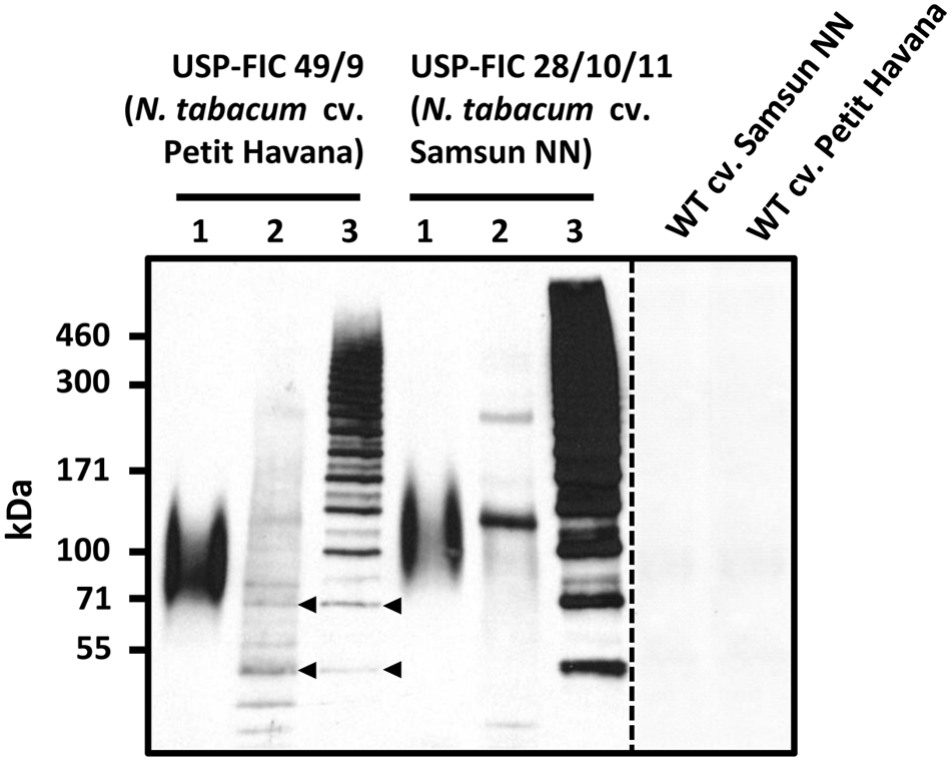
**Intein-mediated formation of high molecular weight FLAG proteins in ripening tobacco seeds.** FLAG precursor proteins under the control of the USP promoter were analyzed in developing tobacco seeds at 18 DAF (1), 21 DAF (2) and in ripe tobacco seeds (3). Extracts from 31.2 seeds were loaded per lane. Seed extracts were separated by gradient SDS-PAGE (4–10% PAA), and FLAG proteins were visualized by immunodetection based on the c-myc tag. DAF, days after flowering; kDa, kilodalton; WT, wild type.

**FIGURE 4:**
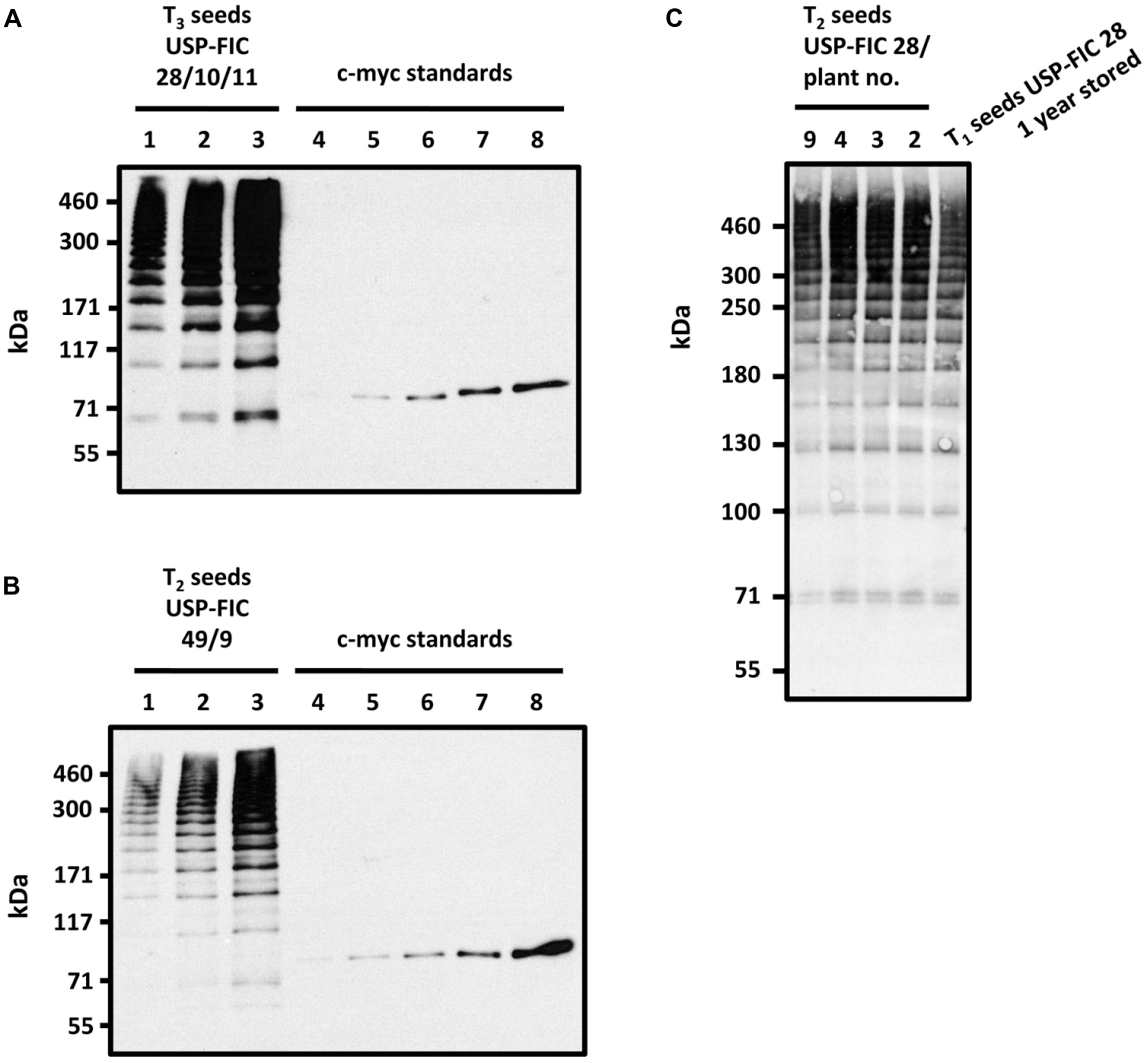
**Stability analysis of multimeric FLAG proteins in transgenic tobacco seeds.** Accumulation of high molecular weight FLAG multimers from T_3_ seeds of USP-FIC 28/10/11 (cv. Samsun NN) stored for 8 weeks at 15°C and 49% humidity **(A)** and T_2_ seeds of USP-FIC 49/9 (cv. Petit Havana) stored under identical conditions. **(B)** Seed extracts were separated by gradient SDS-PAGE (4–10% PAA). Line USP-FIC 28/10/11: 1.4 seeds per lane, (2) 2.8 seeds per lane, (3) 5.6 seeds per lane; Line USP-FIC 49/9: (1) 1.7 seeds per lane, (2) 3.4 seeds per lane, (3) 6.8 seeds per lane. c-myc immunoblot standards: (4) 0.1 ng, (5) 0.25 ng (6) 0.5 ng, (7) 1.0 ng, (8) 2.0 ng of anti-TNF-V_H_H-100xELP. **(C)** Stability of FLAG multimers in the T_1_ seeds of transformed T_0_ plant USP-FIC 28 after 1 year of storage at 15°C and 49% humidity compared to the FLAG accumulation pattern in seed extracts of freshly harvested T_2_ seeds of selected heterozygous USP-FIC 28/plants. Seed extracts were separated by gradient SDS-PAGE (3–10% PAA) with 20 μg total soluble seed protein per lane and visualized by immunodetection based on the c-myc tag. kDa, kilodalton.

A synthetic FLAG gene coding for a monomer of 37.6 kDa (Hauptmann et al., 2013) was cloned into a seed-specific expression vector providing ER retention by providing a signal peptide and the N-terminal KDEL motif (**Figure 1A**). The seed-specific expression was driven by the USP promoter proven for overexpression of transgenic proteins in seeds (Fiedler et al., 1997). The synthetic FLAG protein sequence is based on *N. clavipes* FLAG sequence motifs (Hayashi and Lewis, 2000; Hauptmann et al., 2015). The expression cassette was cloned into a suitable shuttle vector (pCB301-Kan, see Materials and Methods), agrobacteria were transformed and stably transformed tobacco plants were produced by an appropriate protocol (see Materials and Methods). Two different tobacco variants, *N. tabacum* cv. Petit Havana and *N. tabacum* cv. Samsun NN, were transformed. *N. tabacum* cv. Petit Havana plants flower more early and, therefore, seeds ripen also earlier than *N. tabacum* cv. Samsun NN seeds (8 days; **Figure 2B**). We wanted to see if this benefit of shorter seed propagation time influences the accumulation levels and/or multimerization. Among 45 Samsun NN T_0_ transformants 26 showed transgene accumulation and among 55 Petit Havana T_0_ transformants 18 showed transgene accumulation. The different accumulation levels in T_1_ seeds are exemplarily shown in **Figure 1B**. In general, more lines with T_1_ seeds accumulating transgenic proteins comparable to line 28 (nine lines) have been identified in Samsun NN compared to Petit Havana (one line; data not shown). Distinct multimeric bands starting with potential FLAG dimers and ending with multimers above the separation power of a 4–10% polyacrylamid gradient SDS-PAGE (above 500 kDa) are visible, which shows, that intein-based splicing functions well in ripe seeds and that at least native-sized spider silk proteins could be produced. Two lines, USP-FIC 28 (*N. tabacum* cv. Samsun NN) and USP-FIC 49 (*N. tabacum* cv. Petit Havana), were selected as the best high producers from each construct and were further propagated by self-pollination. Equal amounts of seed extract from each of the five sublines were investigated according to the expression of FLAG multimers (**Figure 2**). Multimeric proteins from the monomer molecular weight up to much more than 500 kDa were detected in each lane. The transgene inheritance and the accumulation level were stable in both lines. According to the accumulation level, the best line was a *N. tabacum* cv. Samsun NN line. We analyzed the accumulation level in seeds of the lines USP-FIC 28 (T_3_ seeds) and and USP-FIC 49 (T_2_ seeds) in a semiquantitative manner (see Materials and Methods; **Figure 4**). We applied extract amounts related to seed numbers and calculated the accumulation of transgenic multimers in relation to the fresh weight. We roughly estimated 190 μg FLAG multimers per g seed (fresh weight) for USP-FIC 28 and 20 μg FLAG multimers per g seed (fresh weight) for USP-FIC 49. To learn more about the protein splicing process in developing seeds, we harvested seeds from transgenic plants with different genetic backgrounds and different seed propagation times (see above) during the ripening process to analyze the recombinant protein accumulation. The USP promoter causes the expression of transgenic proteins in tobacco seeds from 10 DAF, with a first maximum at day 17 (Fiedler et al., 1997). Therefore, we selected 18 DAF, 21 DAF and ripe seeds (**Figure 3**), extracted them and analyzed the extracts on a 4–10% polyacrylamid gradient SDS-PAGE and c-myc immunodetection based on extracts from 31.2 seeds per lane independent on plant and age of the seeds to normalize the results according to the fast growing seed protein amount during ripening. In both genetic backgrounds, there was a smear at approximately 100 kDa at 18 DAF and several distinct bands at 21 DAF, but at this time point, they do only partly reflect the expected pattern of different multimerization stages in Petit Havana (monomer, dimer and trimer, labeled in **Figure 3**), whereas in Samsun NN ripening seeds a prominent band at the size of the smear shown in lane 1 is visible.

### Transgenic Tobacco Seeds Containing FLAG Multimers Could be Stably Stored Without Loss of the Transgenic Proteins

One major benefit of seed-based production of functional proteins as antibody fragments is the stability of these proteins in shape and function over a long time at room temperature during seed storage (Fiedler and Conrad, 1995). We wanted to test whether the spider silk multimers are also stable at ambient temperature. T_3_ seeds of USP-FIC 28/10/11 (*N. tabacum* cv. Samsun NN) and T_2_ seeds of USP-FIC 49 (*N. tabacum* cv. Petit Havana) were stored at 15°C and 49% humidity (standard conditions for tobacco seed storage at the Genebank Gatersleben) for 8 weeks, extracted and analyzed (**Figure 4**). For both types of seeds, we showed that the storage of seeds for 8 weeks at ambient temperature did not influence the pattern of multimeric bands. We also stored T_1_ USP-FIC 28 (*N. tabacum* cv. Samsun NN) seeds for 1 year at the ambient conditions mentioned above and analyzed spider silk accumulation in comparison to freshly harvested T_2_ seeds of the same line. Even after this extended storage time, a typical pattern of multimerized bands and a high accumulation level were observed (**Figure 4C**). These results indicate that seeds are the method of choice for stable accumulation of products of complex transgenes, including the capability of long-term storage at moderate conditions.

### Accumulation of Spider Silk Multimers in the ER does not Influence Seed Ripening and Major Seed Protein Content

**FIGURE 5:**
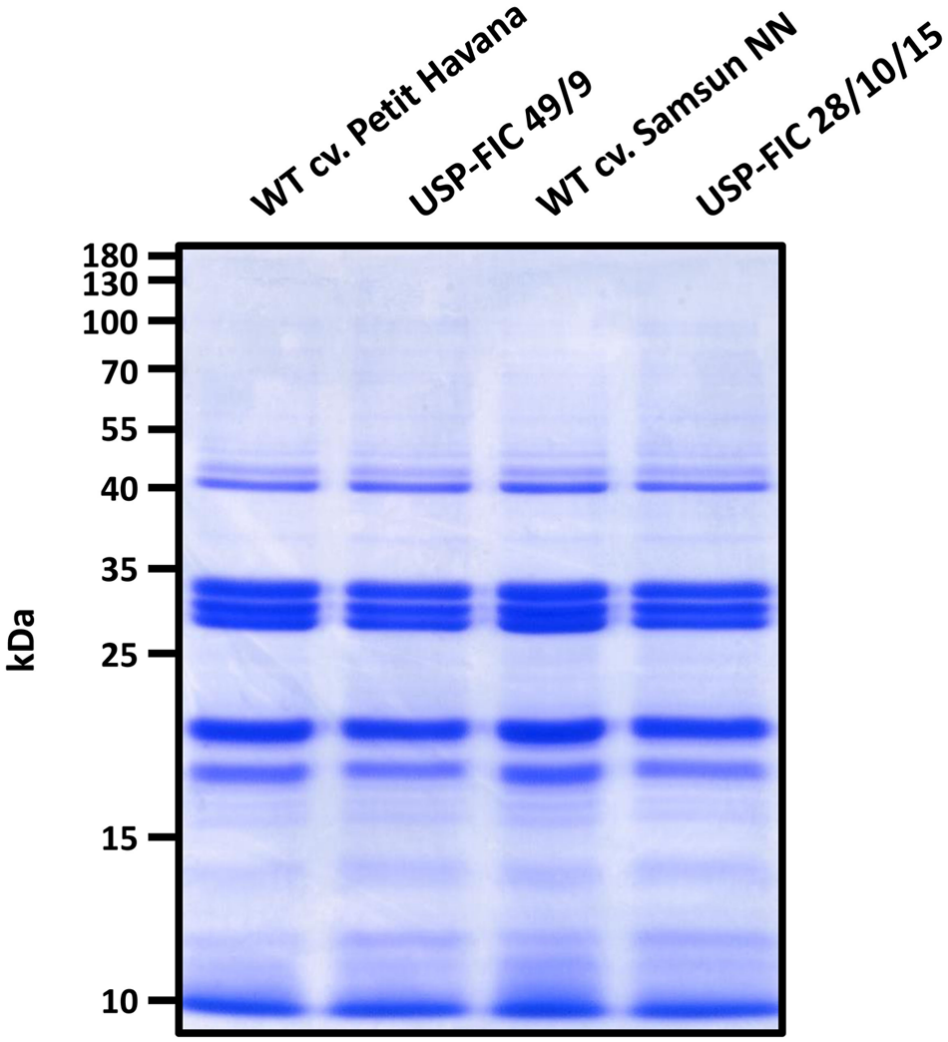
**Analysis of endogenous seed proteins in transgenic tobacco seeds.** Extracts from ripe seeds of two transgenic lines (USP-FIC 28/10/15 and USP-FIC 49/9) and their corresponding wild type cultivars were prepared. Total amounts of 5 μg total soluble protein were separated by SDS-PAGE (12% PAA) and visualized by Coomassie staining.

We did not observe an obvious influence of the spider silk transgene on the development of neither Samsun NN nor Petit Havana lines (**Figure 2B**). High accumulation of anti-hapten scFv in the ER of tobacco seed cells (until 2.6% TSP) did not influence the tobacco seed proteins in ripe tobacco seeds (Phillips et al., 1997). Therefore, we also analyzed the major proteins in ripe T_3_ seeds of the line USP-FIC 28 and ripe T_2_ seeds of the line USP-FIC 49 compared to the seed proteins of their corresponding wild type cultivars. The major seed protein analysis by polyacrylamid gel electrophoresis and Coomassie staining gives no arguments for any influence of the spider silk accumulation to seed development (**Figure 5**).

## Discussion

Seeds can provide stable expression of therapeutic proteins, as shown for several antibodies, antibody derivatives, and vaccines (Stöger et al., 2005). In this paper, we show that large-sized spider silk multimers can be efficiently produced in seeds. We demonstrate stable inheritance and seed-specific expression over three or two generations, respectively, in transgenic lines in two different genetic backgrounds, *N. tabacum* cv. Samsun NN and *N. tabacum* cv. Petit Havana. Whereas *N. tabacum* cv. Samsun NN needs more time to start flowering, the recombinant protein accumulation level in seeds is better than in *N. tabacum* cv. Petit Havana according to the best-expressing plant or according to the general pattern of expressing lines. The seed ripening process itself is not influenced. The patterns of major seed proteins are not different between ripe wild type seeds and ripe transgenic seeds in Samsun NN as well as in Petit Havana (**Figure 5**). The USP promoter has been described as a seed-specific promoter (Bäumlein et al., 1991), but the expression analysis of transgenic tobacco plants by sensitive enzyme activity assays showed minor expression in several other organs and cells (Saalbach et al., 1994). Nevertheless, high mainly seed-specific expression has been shown for recombinant antibodies (Phillips et al., 1997; Floss et al., 2009). The USP promoter is continuously driving the accumulation of transgenic proteins from 10 to 28 DAF (Fiedler et al., 1993, 1997), but this should not negatively influence the final content of transgenic proteins in seeds. As shown in **Figures 1–3**, multimers larger than 460 kDa in size are routinely produced. This is essentially above the known native size of the FLAG protein (Ayoub et al., 2007). The positive influence of the molecular weight on the mechanical properties of plant-produced spider silk proteins has already been demonstrated (Hauptmann et al., 2013; Weichert et al., 2014). Such large-sized spider silk proteins can produce fibers and networks with better mechanical properties by electrospinning as well as materials with superior properties for medical applications (Hauptmann et al., 2013, 2015). Two counteracting facts influence the documentation of this multimerization processes. On the one hand, the c-myc-tag is multimerized together with the FLAG protein, thus causing stronger signals in larger multimers. On the other hand, the efficiency of the electrotransfer process decreases with increasing molecular weight, especially above 200 kDa. The accumulation of FLAG multimers of maximally 190 μg/g fresh weight we roughly estimated fits well into accumulation levels reported for seed expression as 160 μg/g fresh weight for recombinant antibodies in barley seeds (Hensel et al., 2015), 46 μg/g fresh weight for recombinant antibodies in rice grains (Vamvaka et al., 2015) and 6.9 μg/g fresh weight for recombinant antibodies in tobacco seeds (Floss et al., 2009). Leaf expression of a recombinant antibody in tobacco driven by a ubiquitous promoter at optimized growth conditions was about 45 μg/g fresh weight (Sack et al., 2015). Larger scale production of seeds, at best in protein-rich seeds such as legumes, combined with the development of a suitable down-stream process can provide enough material for the directed enrichment of fractions above 200 kDa, reflecting the native size. The process of *trans*-splicing requires reassociation of the intein fragments before splicing occurs (Topilina and Mills, 2014). Whereas the intein-based self-excision and ligation is expected to occur immediately after the translation and folding (Aranko et al., 2014), the reassociation may need more time, and we expect a concentration-dependence of this process. In addition, exteins can chemically or structurally influence the active site of inteins (Eryilmaz et al., 2014). During the formation of the multimers, several reassociation and splicing events occur on the same protein chain but not necessarily at the same time. This may explain the smear of proteins with slightly differing molecular weights at 18 DAF (**Figure 3**). At 21 DAF, distinct bands occur, but the expected pattern of multimerization is only visible in ripe seeds. In the faster developing Petit Havana seeds at 21 DAF bands corresponding to monomers, dimers and trimers were identified (**Figure 3**). Generally, the accumulation level per seed is much lower at 18 and 21 DAF than in ripe seeds. In pre-experiments, the construct was transiently expressed in *N. benthamiana* by the co-expression of a seed-specific transcription factor (FUSCA 3) binding to elements in the USP promoter (Mönke et al., 2004). Even 6 days after treatment with agrobacteria, strong expression, distinct bands and an expected pattern are visible (Supplementary Figure S1). These two observations are arguments that a certain transgene accumulation level is necessary for the intein-based protein splicing *in planta*. This level is provided by continuous promoter activity combined with stable accumulation in the ER provided by ER retention (Fiedler et al., 1997). ER retention has been proven for the accumulation of different spider silk proteins (Scheller et al., 2001; Hauptmann et al., 2013; Weichert et al., 2014). Yang et al. (2005) analyzed the accumulation of a synthetic spider silk dragline protein of 64 kDa in the apoplast, the vacuole and the ER lumen in *Arabidopsis* seeds and leaves. The highest accumulation levels have been reported for the ER lumen. The authors recommend seed-specific expression and ER targeting for plant-based spider silk protein expression as a result of their *Arabidopsis* experiments. We showed here, that this holds true also for spider silk multimers of native size in tobacco seeds. One of the goals of the experiments presented here was to test whether spider silk multimers in seeds are stable at room temperature without a decline in protein accumulation and without a change in the multimerization pattern. The data presented here show stability in the amount and multimerization pattern for 8 weeks storage at 15°C and 49% humidity. In addition, long-term storage of T_1_ seeds for 1 year at these conditions resulted in a completely identical size distribution of the multimers and clear bands; thus, no indications of proteolysis were found. Further work should include the development of transgenic lines in protein-rich seeds. Here, the suitability of the USP promoter and ER retention has already been proven (Zimmermann et al., 2009). The high stability in seeds is a major advantage for the development of a suitable down-stream process.

## Author Contributions

Conceived and designed the experiments: NW, VH, and UC. Plasmid construction and transient tests: VH. Performed all other experiments: NW and CH. Analyzed the data: NW, CH, and UC. Wrote the paper: NW, VH, and UC.

## Conflict of Interest Statement

The authors declare that the research was conducted in the absence of any commercial or financial relationships that could be construed as a potential conflict of interest.
