# Supplementary material for: Seed-Specific Expression of Spider Silk Protein Multimers Causes Long-Term Stability
Source: Front Plant Sci. 2016 Jan 28;7:6. doi: 10.3389/fpls.2016.00006 (PMC4729946; doi:10.3389/fpls.2016.00006)
Supplement: Supplementary file 1 [file Image_1.PDF]

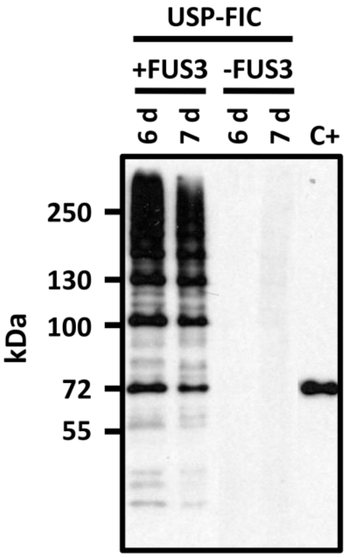

Supplementary Figure. Transient expression of FLAG multimers in *Nicotiana benthamiana* leaves. Expression of the USP-FIC construct in leaves was driven by co-expression of the seed-specific transcription factor FUSCA3, which binds to elements in the USP promoter sequence (Mönke et al., 2004). The transient transformation was performed as described by Phan et al. (2013). Six days after treatment with agrobacteria, a strong and expected expression pattern of FLAG multimers was observed. Leaf extracts were separated by gradient SDS-PAGE (3–12 % PAA), and FLAG proteins were visualized by immunodetection based on the c-myc tag. c+, 1 ng of anti-TNF-V_HH-100xELP (Conrad et al., 2011)

References

Conrad, U., and Fiedler, U. (1998): Compartment-specific accumulation of recombinant immunoglobulins in plant cells: essential tool for antibody production and immunomodulation of physiological functions and pathogen activity. *Plant Mol Biol* 38, 101-109. doi 10.1023/A:1006029617949.

Mönke, G., Altschmied, L., Tewes, A., Reidt, W., Mock, H.P., Bäumlein, H., and Conrad, U. (2004). Seed-specific transcription factors ABI3 and FUS3: molecular interaction with DNA. *Planta* 219**,** 158-166. doi: 10.1007/s00425-004-1206-9.

Phan, H.T., Pohl, J., Floss, D.M., Rabenstein, F., Veits, J., Le, B.T., Chu, H.H., Hause, G., Mettenleiter, T., and Conrad, U. (2013). ELPylated haemagglutinins produced in tobacco plants induce potentially neutralizing antibodies against H5N1 viruses in mice. *Plant Biotechnol J* 11**,** 582-593. doi: 10.1111/pbi.12049.
